# Airway management education: simulation based training versus non-simulation based training-A systematic review and meta-analyses

**DOI:** 10.1186/s12871-017-0313-7

**Published:** 2017-02-01

**Authors:** Yanxia Sun, Chuxiong Pan, Tianzuo Li, Tong J. Gan

**Affiliations:** 1 Department of Anesthesiology, Beijing Tong Ren Hospital, Capital Medical University, Beijing, China; 2 Department of Anesthesiology, Beijing Shi Ji Tan Hospital, Capital Medical University, Beijing, China; 3 Department of Anesthesiology, Stony Brook University, Stony Brook, USA

**Keywords:** Simulation, Airway management, Training

## Abstract

**Background:**

Simulation-based training (SBT) has become a standard for medical education. However, the efficacy of simulation based training in airway management education remains unclear.

**Methods:**

The aim of this study was to evaluate all published evidence comparing the effectiveness of SBT for airway management versus non-simulation based training (NSBT) on learner and patient outcomes.

Systematic review with meta-analyses were used. Data were derived from PubMed, EMBASE, CINAHL, Scopus, the Cochrane Controlled Trials Register and Cochrane Database of Systematic Reviews from inception to May 2016. Published comparative trials that evaluated the effect of SBT on airway management training in compared with NSBT were considered. The effect sizes with 95% confidence intervals (CI) were calculated for outcomes measures.

**Results:**

Seventeen eligible studies were included. SBT was associated with improved behavior performance [standardized mean difference (SMD):0.30, 95% CI: 0.06 to 0.54] in comparison with NSBT. However, the benefits of SBT were not seen in time-skill (SMD:-0.13, 95% CI: −0.82 to 0.52), written examination score (SMD: 0.39, 95% CI: −0.09 to 0.86) and success rate of procedure completion on patients [relative risk (RR): 1.26, 95% CI: 0.96 to 1.66].

**Conclusion:**

SBT may be not superior to NSBT on airway management training.

## Background

Airway management is often a life-saving procedure for patients. However, it may be difficult for many health-care providers to gain enough experience to become and remain expert in airway management based solely on their clinical experience [1]. Thus, it is helpful for them to receive additional training in airway management beyond their clinical experience.

There are several methods of medical education on airway management training. Simulation based training (SBT) has gained much attention as it may improve patients safety and increase learner competence [2, 3]. Systematic reviews show that simulation based training provides consistent benefits in medical education. Non- -simulation based trainings (NSBT) have also been used for airway management education including lecture, video, discussion, problem based learning, and clinical observation. There are several studies examining the efficacy of SBT in comparison with NSBT on airway management education. However, the results of those studies are conflicting. The objective of this up-to-date systematic review is to evaluate all the published evidence to compare the effectiveness of airway management training using either SBT or NSBT on learner and patient outcomes. Our primary hypothesis is that SBT is superior to NSBT on airway management training.

## Methods

We followed PRISMA guideline in reporting this systematic review and meta-analysis [4]. A review protocol was written prior to conducting this study.

### Inclusion criteria

We considered all published comparative trials that evaluated the effect of simulation on airway management training in comparison with NSBT. We used the following inclusion criteria to select the pool of eligible studies:

1. Feature SBT as an educational intervention involving one or more of following modalities: partial-task trainer (commercially available, homemade trainers, or animal models), high-fidelity mannequins, virtual reality, or computer software [5]
2. Feature NSBT as a comparison group
3. In single-task or multitask course which included training for airway management technique (e.g. direct laryngoscope and/or intubation (DL), bag-mask-ventilation (BMV), flexible laryngoscope or bronchoscope (FL), supraglottic airway management, cricoids pressure and surgical airway).
4. Assessment of learner and/or patient outcomes.

Data from letters, case reports, reviews or abstracts were excluded.

### Search strategy

A systematic search of PubMed, EMBASE, CINAHL, Scopus, the Cochrane Controlled Trials Register and Cochrane Database of Systematic Reviews from inception to May 2016, was performed to identify published potential trials. The search strategy was developed using following search terms: *airway; fiberoptic, fiberscope; bronchoscopy; laryngoscopy; intubation; supraglottic, laryngeal mask, combitube; cricoids pressure; bag-mask-ventilation; cricothyroidotomy, surgical airway*. These terms were searched as subject headings, medical subject headings, and text words where appropriate. We combined these using the Boolean operator “and” with education terms: *training; education; learning; teaching; and teach*. No language restriction was placed on our search. To maximize the sensitivity of our search, we did not limit our search to terms related to simulation or study type. The reference lists of all eligible publications and reviews were scanned to identify additional relevant studies. Two authors screened and reviewed independently all titles and abstracts for eligibility. For abstracts that did not provide sufficient information to determine eligibility, full-length articles were retrieved. Disagreement on inclusion or exclusion of articles was resolved by consensus.

### Data extraction and quality assessment

Studies were reviewed and data extracted independently by two authors using a pre-designed standard form. The following data points were extracted: 1) simulation modality, 2) trainee characteristics, 3) airway management techniques, 4) type of study design, 6) method of assessment, 7) learning outcomes, including time-skill (time to complete task), behavior performance and knowledge, 8) learner reaction (i.e. satisfaction, interest and confidence), 9) patient clinical outcomes (i.e. success rate of procedure completion on patients and complications of airway management). Attempts were made to contact the authors for missing data. If detailed information was not received, the study was excluded from the current meta-analysis.

To assess methodological quality, we used elements of the Medical Education Research Study Quality Instrument (MERSQI) [6]. The MERSQI is a10-item tool for the evaluation of quality of medical education studies, examining domains in study design, sampling, validity of assessments, data analysis, and outcomes. Two authors assessed the quality of included studies independently; disagreements were settled by consensus.

### Statistical analysis

Continuous data were pooled as standard mean differences (SMD) with 95% confidence interval (CI). A SMD of 0.2 is considered small effect size; 0.5, moderate; and 0.8, large. Dichotomous data were analyzed using risk ratio (RR) with 95% CI. The Cochrane chi-square Q statistics and I^2^were used to assess heterogeneity across studies, which determined the appropriate use of either fixed-effects or random-effects model. Heterogeneity was considered as a *P*-value < 0.05 or I^2^ > 25% [7]. A random-effects model was used if heterogeneity was considered.

We conducted a sensitivity analysis by restricting the analysis to randomized controlled trials (RCTs). Pre-specified subgroup analyses were also conducted based on type of airway management techniques. In addition, publication bias (failure to publish negative studies) was evaluated using the Begg’s funnel plots, which is a scatter plot of magnitude of effect size against a measure of its precision. We performed the trim and fill procedure to further assess potential effects of publication bias. Analyses were conducted using RevMan 5.1 and Stata/SE 10.0 (College Station, TX, USA).

## Results

**Fig. 1:**
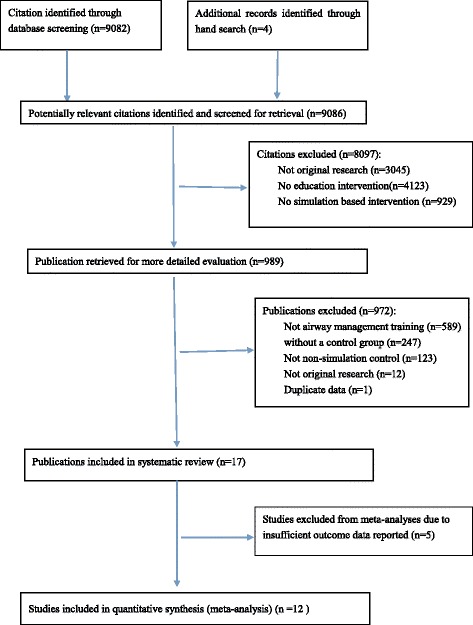
Flowchart of study selection

We identified 9086 articles for title and abstract screening. After applying inclusion and exclusion criteria, we excluded 8097 articles because they were not original research, did not involve medical learner, nor evaluating the effect of SBT. The remaining subset of 989 articles were gathered for further review. This group was evaluated in detail by each author to reach consensus on whether the articles met the inclusion criteria described above until full consensus was reached. Of this group, 972 articles were excluded because of duplication of published data, not relevant for airway management training, not featuring NSBT as a control group or published in letter or abstract. A total of 17 articles were finally considered for this review (Fig. 1).

### Study characteristics

The characteristics of the 17 studies included in this systematic review were listed in Table 1. Majority trainees were medical students or physicians with limited related airway management experience from various specialties (Table 1). Median sample size of included studies was 60 (range 6–245). Thirteen out of 17 studies (76%) [8–20] were RCTs, whereas four studies (24%) [21–24] were of non-randomized two-group study design. The median MERSQI scores were 13.5 (range 9.5–16).

**Table 1:** Characteristics of included studies

Trials	Number of learners	Study design	Type of participants/Specialties	Type of NSBT	Type of SBT	Airway management techniques	Outcomes	MERSQI
Ovassapian A (1998)^a^ [8]	32	RCT	Residents/Surgery and anesthesiology	Instructor demonstration	Unspecified model simulator	FL	SR(p), BP, complications	14
Naik VN (2001)^a^ [9]	24	RCT	Residents/Anesthesiology and internal medicine	lecture	Partial-task simulator	FL	SR(p), TS, BP complications	16
OST D (2001)^a^ [10]	6	RCT	First –year fellows/Pulmonary	Clinical observation	Partial-task simulator	FL	TS, BP complications	13
Modell JH (2002)^a^ [11]	40	RCT	Students/Veterinary	Self study	Virtual reality with HPS	DL	ES	11.5
Morgan PJ (2002)^a^ [12]	144	RCT	Final-year medical students	Video	Virtual reality with HPS	DL	BP, ES, LR	12.5
Multak N (2002)^a^ [21]	56	2NR	Students/Physician assistant	Video and discussion	Virtual reality with HPS	DL	ES	9.5
Hall RE (2005)^a^ [13]	36	RCT	Second-year students/Paramedic	Clinical observation	Virtual reality with HPS	DL	SR(p), Complications	12.5
Chen JS (2006)^a^ [14]	20	RCT	Novice bronchoscopists/NR	Video and clinical observation	Partial-task simulator	FL	LR	10
Quigley P (2007) [22]	70	2NR	Nurse and doctors/Emergency	Self study	Partial-task simulator	CP	SR(s)	11.5
Kory P (2007) [23]	64	2NR	residents/Internal medicine	Clinical observation	Virtual reality with HPS	BMV	BP	12
Youngquist ST (2008) [24]	245	2NR	Students/Paramedic	Lecture/video	Unspecified model simulator	BMV, DL	LR, SR(s)	13.5
Hallikainen J (2009) [15]	46	RCT	Fourth year medical students	Clinical observation	Virtual reality with HPS	DL	SR(s)	13.5
Gaies MG (2009)^a^ [16]	38	RCT	Interns/Pediatric	Clinical observation	Unspecified model simulator	BMV	SR(s), BP	14
Wenk M (2009)^a^ [17]	33	RCT	Fourth-year medical students/NR	Problem-based discussion	Virtual reality with HPS	DL	TS, BP, ES, LR	14.5
Campos JH (2011)^a^ [18]	56	RCT	Fellows and faculty with limited related experience/Anesthesiology	video	Partial-task simulator	DL	SR(P), TS, complications	15
Smith ME (2014)^a^ [19]	36	RCT	Final-year medical students and residents/NR	Lecture and video	Partial-task simulator	FL	TS, BP, complications	15
Jayaraman V (2014) [20]	19	RCT	Medical students/Surgery	Video	Partial-task simulator	Cricothyroidotomy	TS, BP, ES, LR, complications	14

*NSBT* non-simulation based training, *SBT* simulation based training, *MERSQI* Medical Education Research Study Quality Instrument

Study design: *RCT* randomized controlled trial, *2NR* non-randomized two-group study design

Type of participants: *NR* not reported

Simulator: *HPS* high fidelity simulator

Airway management technique: *BMV* bag-mask-ventilation, *CP* cricoid pressure, *DL* direct laryngoscopy and/or intubation, *FL* flexible laryngoscopy or bronchoscopy

Outcomes: *BP* behavior performance, *ES* examination score, *LR* learner reactions, *SR(p)* success rate on patients, *SR(s)* success rate on simulators, *TS* time-skill

^a^studies included in meta-analysis

Seven studies [11–13, 15, 17, 21, 23] used virtual reality in addition to a high-fidelity simulator model, seven studies [9, 10, 14, 18–20, 22] used partial-task simulators and three [8, 16, 24] used unspecified simulator models. Specific techniques of airway management evaluated included DL [11–13, 15, 17, 18, 21, 24], FL [8–10, 14, 19], BMV [16, 23, 24], cricoids pressure [22] and surgical airway [20].

### Meta –analyses

#### Learning outcomes: time-skill, behavior performance and knowledge

**Fig. 2:**
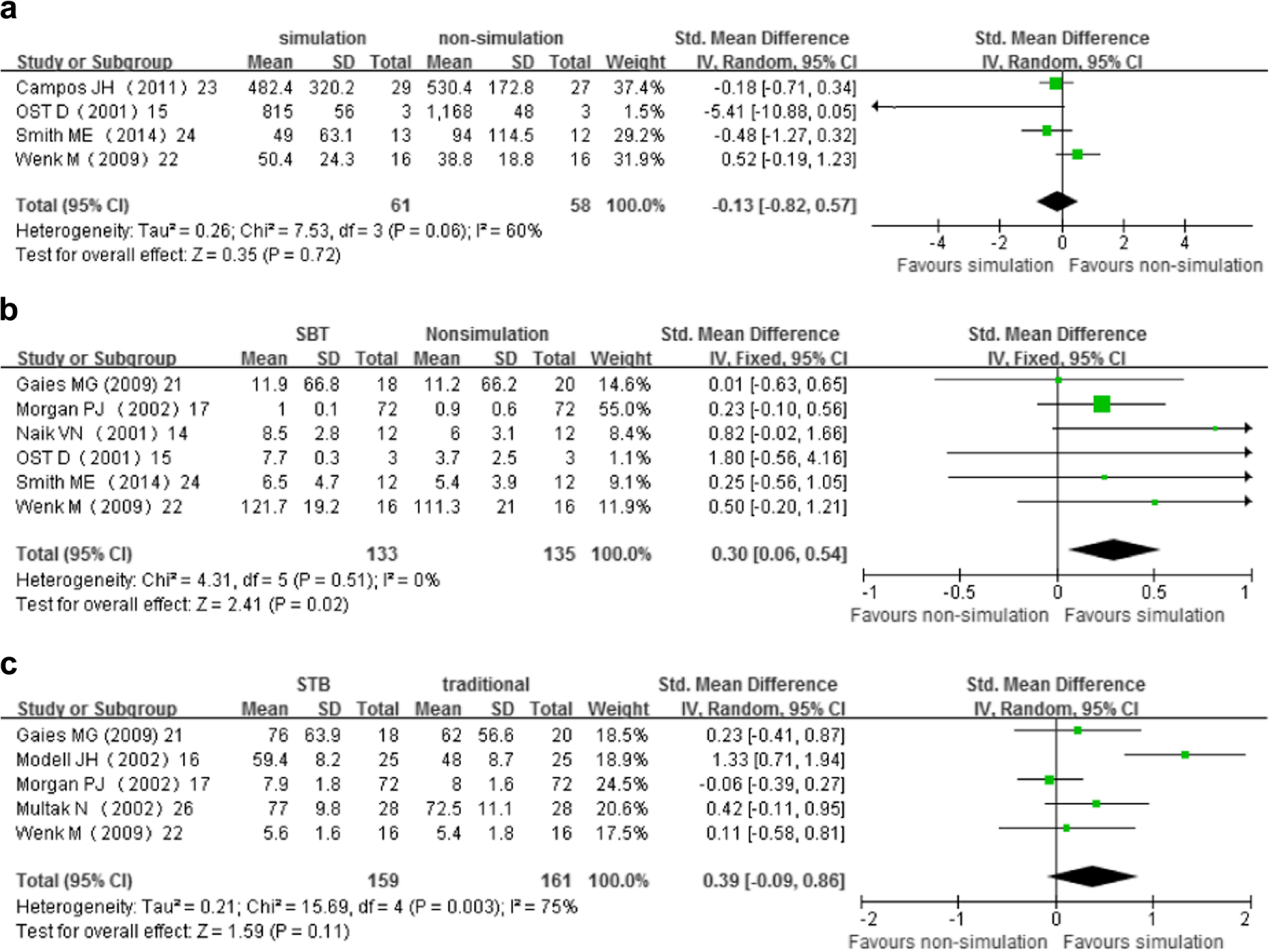
Meta-analyses of the effect of simulation-based training compared with non-simulation instruction on learning outcomes. Panel 2**a** shows the standardized mean difference in time-skill, random-effects model. Panel 2**b** shows the standardized mean difference in behavior performance, fixed-effects model. Panel 2**c** shows the standardized mean difference in examination score, random-effects model

Six studies [9, 10, 17–20] presented data on time to complete task. Of these, two studies [9, 20] were not included in the meta-analysis because data were reported as median with interquartile range. Both of those two studies found that time to complete task was faster in simulation group than that in control groups. However, pooled estimates of the remaining four studies [10, 17–19] showed no significant difference between SBT and NSBT groups. Pooled random-effects SMD was −0.13 (95% CI: −0.82 to 0.57) (Fig. 2). Trim-and-fill analyses revealed no trimming performed and data unchanged.

Six studies [9, 10, 12, 16, 17, 19] reported suitable data on behavior performance. SBT had favorable effect on behavior performance in comparison with NSBT controls. Pooled fixed-effects SMD was 0.30 (95% CI 0.06 to 0.54) (Fig. 2). However, the effect size was small. Trim-and-fill analyses showed a revised pooled SMD of 0.27 (0.03 to 0.52).

Six studies [11, 12, 16, 17, 20, 21] used written examination scores to evaluate knowledge acquisition. One study [20] reported data as median with interquartile range, therefore it could not be included in the meta-analysis. This study showed that the SBT and NSBT groups did not differ on knowledge acquisition. Data from the remaining five studies [11, 12, 16, 17, 21] were analyzed. The pooled random-effects SMD of 0.39 (95% CI:−0.09 to 0.86) showed no significant difference between SBT group and NSBT group (Fig. 2). The trim-and-fill analysis showed no trimming performed and the pooled SMD was not changed.

After excluding 4 studies [21–24], the overall results for learning outcomes were not affected by sensitivity analyses of RCTs (Table 2). The results of subgroup analyses based on the type of airway management technique were also listed in Table 2. Only FL and DL training have enough studies for subgroup analyses. Subgroup analyses found that SBT was not associated with significant improvement in time-skill and knowledge acquisition for both DL and FL training. The benefit of SBT was only seen in learner behavior performance for FL education. (SMD: 0.59, 95% CI: 0.03 to 1.16).

**Table 2:** Results of subgroup analyses and sensitivity analysis

Subgroups and sensitivity analysis	Time-skill SMD and 95%CI [number of studies]	Behavior performance SMD and 95%CI [number of studies]	Examination scores SMD and 95%CI [number of studies]
Airway management techniques			
FL	−2.17 (−6.77,2.42) [*n* = 2]	0.59 (0.03,1.16) [*n* = 3]	0.43 (−0.16,1.03) [*n* = 4]
DL	0.13 (−0.56,0.81) [*n* = 2]	0.28 (−0.02,0.58) [*n* = 2]	NA
Sensitivity analysis of RCTs	−0.13 (−0.82, 0.57) [*n* = 4]	0.30 (0.06, 0.54) [*n* = 6]	0.38 (−0.24, 1.00) [*n* = 4]

*CI* confidence interval, *SMD* standardized mean difference, *DL* direct laryngoscopy or/and intubation, *FL* flexible laryngoscopy or bronchoscopy, *RCTs* randomized controlled trials, *NA* not available

#### Learner reaction: confidence, interest and satisfaction

SBT also has favorable effects on learner interest and satisfaction. Pooled random-effects SMD was 0.63 [95%CI 0.32 to 0.95, *p* = 0.0003, number of studies (n) = 2 [12, 14]] and 0.58 [95%CI 0.27 to 0.90, *p* < 0.0001, *n* = 2 [12, 14]] for learner interest and satisfaction, respectively.

One study [17] reported measures of self-assessment of confidence. The confidence scores were 21.0 (3.2) in SBT group and 19.4 (1.9) in the NSBT group, indicated that learner in SBT group felt more confident than those in NSBT group.

#### Patient clinical outcomes

Six studies [8–10, 13, 18, 19] assessed learner skill acquisition on patients or volunteers. Four studies [8, 9, 13, 18] provided success rate of procedure completion. The risk ratio (RR) was 1.26 (95% CI: 0.96 to 1.66), indicating no significant difference between SBT and NSBT groups.

Seven studies [8–10, 13, 18–20] reported procedure related complications. Pooling of those data across the trials was deemed impossible due to heterogeneous reporting outcomes and methods. Four studies [8–10, 18] reported that no patients in both two groups suffered any significance adverse effects during procedure. Three studies [13, 19, 20] reported procedure-related complications (e.g. pain, bleeding, esophageal intubation) and found no statistically significant differences in complications between SBT and NSBT groups.

#### Skill retention

Three studies [16, 22, 24] evaluated and demonstrated retention of skills with repeated testing 4 weeks or 8 months later. The airway management techniques included BMV, DL and cricoids pressure. All of these studies showed that the skills decay significantly in both SBT and NSBT groups and the between-group differences were no longer evident at follow-up assessment. One study [24] found that DL skills drop off more significantly than BMV skills.

## Discussion

There are high expectations associated with SBT since it could apply knowledge in a hands-on approach and offer a venue for problem solving in real-life situation without patient risk or time constraints [25–27]. The previous systematic reviews found that SBT for airway management training was associated with improved outcomes compared with NSBT [28]. Our systematic review focused on the comparative effectiveness between SBT and NSBT and included seven more studies [10, 14, 16, 19, 20, 22, 23]. We found that SBT slightly improved performance behaviors and increase learner’s satisfaction and interest when compared with NSBT. However, benefits of SBT were not seen in time-skill and knowledge acquisition. We also conducted sensitivity and subgroup analyses to provide further robustness to the data. Again, our subgroup analyses failed to demonstrate a significant benefit of SBT in time-skill and knowledge acquisition for both FL and DL training.

Our findings supported the previous evidence that SBT are enjoyable and attractive instruments for airway management training [29]. However, clinical or knowledge advantage remains a significant concern to justify the implementation of simulation in a medical program. In the current systematic review, we could not demonstrate the benefit of any group in time-skill improvement and knowledge acquisition. Although trainees in SBT group showed a significant improvement in performance behaviors, this did not translate into increased success rate in clinical setting. Moreover, three of included studies showed that the skills decay significantly in both SBT and NSBT groups and the between-group differences were no longer evident at follow-up assessment. Those findings demonstrate that both SBT and NSBT for airway management provided similar effects on short-term learning and clinical skills improvement, as well as skill retention.

Several limitations of this review are note worthy. First, our analysis revealed high inconsistency between studies, reflecting variation in instructional design, learner groups, NSBT methods and outcome measures. Secondly, some of the included studies had methodological limitations or failed to describe clearly the context, instructional design, or outcomes; and these deficits limit the strength of our inferences. Some studies could not be included in the pooled analyses because of missing data, despite numerous attempts to contact the authors for more information. Thirdly, only one-third of the included studies measured outcomes on real clinical setting and three studies provided data on skill retention, thus limiting our ability to comment on translation of outcomes from the simulated environment to the real life clinical environment. Last, pooling effect sizes across study designs is problematic. We have therefore provided results for our meta-analyses, stratified by study design. Results from meta-analyses of RCTs remain consistent.

## Conclusion

This meta-analysis , within limitations of the existing data and of the analytic approaches used, shows that SBT is associated with improving learner behaviour performance and increasing learner interest and satisfaction. But no significant effect of SBT on time skill and knowledge acquisition for airway management was found. Further well-designed studies are needed to address this issue.

## Abbreviations

BMV: Bag-mask-ventilation
CI: Confidence interval
DL: Direct laryngoscope and/or intubation
FL: Flexible laryngoscope or bronchoscope
MERSQI: Medical Education Research Study Quality Instrument
NSBT: Non -simulation based training
RCT: Randomized controlled trial
RR: Risk ratio
SBT: Simulation based training
SMD: Standardized mean difference
